# Chloroform in Traumatic Tetanus

**Published:** 1849-02

**Authors:** 


### SURGERY.

*Chloroform in Traumatic Tetanus.*—A student of medicine at Columbia, Tenn., writing to a friend in this city, says: "A young physician in this town, about a year since, gave chloroform to a person previously to extracting a tooth, and, for some reason, very alarming symptoms followed. Since that time it was not used here until the day before yesterday, when on consultation, finding it highly recommended in Prof. Yandell's introductory lecture, it was determined to give it in a case of traumatic tetanus. Large doses of opium, and other antispasmodics had been previously tried without effect. The experiment was witnessed by most of the physicians in Columbia, and painful apprehensions were felt for the result. Under the influence of the anæsthetic the patient fell into an easy and refreshing slumber, a pleasant smile resting upon his countenance which before was haggard. His muscles relaxed; his mouth was easily opened; his pulse improved; the spasms ceased. When he awoke, the tetanic symptoms returned, but not with the same violence. It was administered a second time after an interval of several hours, but did not entirely overcome the muscular contractions. It was not afterwards repeated, and the patient eventually died."—*Western Journal.*

